# Transcriptional landscape of human neuroblastoma cells in response to SARS-CoV-2

**DOI:** 10.1186/s12868-022-00728-6

**Published:** 2022-07-06

**Authors:** Rui-Cheng Yang, Kun Huang, Hui-Peng Zhang, Liang Li, Chen Tan, Huan-Chun Chen, Mei-Lin Jin, Xiang-Ru Wang

**Affiliations:** 1 State Key Laboratory of Agricultural Microbiology, The Cooperative Innovation Center for Sustainable Pig Production, College of Veterinary Medicine, Huazhong Agricultural University, Wuhan, Hubei 430070 China; 2 Key Laboratory of Preventive Veterinary Medicine in Hubei Province, Wuhan, Hubei 430070 China

**Keywords:** SARS-CoV-2, SK-N-SH cells, RNA sequencing, Neuroinflammation

## Abstract

**Background:**

Severe acute respiratory syndrome coronavirus 2 (SARS-CoV-2) is highly contagious, and the neurological symptoms of SARS-CoV-2 infection have already been reported. However, the mechanisms underlying the effect of SARS-CoV-2 infection on patients with central nervous system injuries remain unclear.

**Methods:**

The high-throughput RNA sequencing was applied to analyze the transcriptomic changes in SK-N-SH cells after SARS-CoV-2 infection. Gene Ontology and Kyoto Encyclopedia of Genes and Genomes analyses were performed to identify the functions of differentially expressed genes and related pathways.

**Results:**

A total of 820 mRNAs were significantly altered, including 671 upregulated and 149 downregulated mRNAs (showing an increase of ≥ 2-fold or decrease to ≤ 0.5-fold, respectively; p ≤ 0.05). Moreover, we verified the significant induction of cytokines, chemokines, and their receptors, as well as the activation of NF-κB, p38, and Akt signaling pathways, in SK-N-SH by SARS-CoV-2.

**Conclusions:**

To our knowledge, this is the first time the transcriptional profiles of the host mRNAs involved in SARS-CoV-2 infection of SK-N-SH cells have been reported. These findings provide novel insight into the pathogenic mechanism of SARS-CoV-2 and might constitute a new approach for future prevention and treatment of SARS-CoV-2-induced central nervous system infection.

**Supplementary Information:**

The online version contains supplementary material available at 10.1186/s12868-022-00728-6.

## Background

Severe acute respiratory syndrome coronavirus 2 (SARS-CoV-2) caused and still causing the well spread coronavirus disease 2019 (COVID-19) pandemic globally [1]. SARS-CoV-2 spreads through the mucus membrane of both the upper and lower respiratory tracts, causes cytokine storm and generates a series of immune responses [2]. Coronaviruses belong to a diverse group of zoonotic, enveloped RNA viruses with positive sense polarity, which includes Middle East respiratory syndrome (MERS), SARS-CoV, and SARS-CoV-2; these viruses are assigned to the Orthocoronavirinae subfamily of the Coronaviridae family [3]. The typical symptoms of COVID-19 include dry cough, fever, fatigue, headache, rhinobyon, diarrhea, and myalgia. Severe patients display acute respiratory distress syndrome (ARDS), metabolic acidosis, septic shock, coagulation dysfunction, and multiple organ dysfunction syndrome [4]. Several studies showed that SARS-CoV-2 enters host cells through interacting with the spike glycoprotein and angiotensin-converting enzyme 2 (ACE2) [5] and priming spike glycoprotein by engaging the cellular serine protease TMPRSS2 [6]. However, the underlying pathogenic mechanism of SARS-CoV-2 requires further study.

According to the pathological findings from autopsies and biopsies, SARS-CoV-2 not only affects the respiratory system, but is also a potential threat to the digestive, urogenital, circulatory, and central nervous systems (CNS) [7]. Case analysis indicated that SARS-CoV-2 can infiltrate the CNS, and 36.4% of COVID-19 patients have neurological symptoms [8]. SARS-CoV-2 infection has also been found to cause viral encephalitis, and the SARS-CoV-2 genome has been detected in cerebrospinal fluid [9]. Meanwhile, autopsy reports indicate encephaloedema and partial neuronal degeneration in deceased patients [10]. However, as patients are often in a state of sedation, more neurological disorders in patients with severe COVID-19 might not be detected. Previous studies have shown that other coronaviruses may invade peripheral nerve terminals and then enter the CNS through the trans-synaptic route [11]. To date, there is sparse direct evidence of the neurotropism of SARS-CoV-2; thus, investigation into the mechanisms and etiology underlying the interplay between SARS-CoV-2 and neurons is urgently required.

Knowledge of SARS-CoV-2 neurotropism and the potential mechanisms of CNS invasion is important for a better understanding of COVID-19 diagnosis, treatment, and prognosis. Here, we applied RNA sequencing (RNA-Seq) and bioinformatics approaches to identify potential host mRNAs active in human neuroblastoma cells (SK-N-SH) upon SARS-CoV-2 infection. Our findings suggest that SARS-CoV-2 has a certain pathogenic effect on SK-N-SH cells, shedding new light on the neuroinflammatory responses caused by SARS-CoV-2 infection.

## Methods

### Viruses and cell lines

SARS-CoV-2 strain Wuhan-Hu-1 (NC_045512) was obtained from the Wuhan Institute of Virology, Chinese Academy of Sciences. *Chlorocebus sabaeus* (Green monkey) VeroE6 (female, RRID:CVCL_YQ49) were purchased from American Type Culture Collection (id: ATCC CRL-1586). VeroE6 cells were cultured in Dulbecco’s modified Eagles medium (DMEM) supplemented with 10% fetal bovine serum (FBS) at 37 °C in a humidified CO_2_ incubator. The SARS-CoV-2 virus stocks were prepared on Vero cells and 50% tissue culture infective doses (TCID_50_) were calculated using the Reed-Muench formula [12]. All experiments involving live viruses were performed in a biosafety level 3 (BSL3) facility in the Huazhong Agricultural University.

The human neuroblastoma cell line SK-N-SH (ATCC^®^ HTB-11) was obtained from Procell Life Science & Technology Co., Ltd (Wuhan, China). SK-N-SH cells were routinely grown in minimum essential medium (MEM) supplemented with 10% FBS. The cells were grown at 37 °C in a humidified atmosphere containing 5% CO_2_.

### SARS-CoV-2 infection of SK-N-SH cells

The confluent SK-N-SH monolayer grown in 12-well plates was washed three times with serum-free MEM before infection at a multiplicity of infection (MOI) of 1. After 1 h of virus adsorption at 37 °C and 5% CO_2_, cultures were washed three times with serum-free MEM to remove unbound virus. The cells were cultured in 2% FBS MEM at 37 °C with 5% CO_2_. Finally, cells were washed three times with pre-chilled phosphate-buffered solution (PBS) at the indicated time points and subjected to RNA extraction using TRIzol reagent (Invitrogen, Carlsbad, CA, USA) or RIPA buffer with phosphatase inhibitor cocktail (MedChemexpress, Monmouth, NJ, USA) for western blot analysis.

### RNA-seq and bioinformatics analysis

A total of 1 µg RNA per sample was used as the input material for library preparation. The mRNA was purified from total RNA using poly T oligo-attached magnetic beads. Sequencing libraries were generated from the purified mRNA using the VAHTS Universal V6 RNA-Seq Library Kit for MGI (Vazyme, Nanjing, China) following the manufacturer’s recommendations with unique index codes. The library quantification and size were assessed using a Qubit 3.0 Fluorometer (Life Technologies, Carlsbad, CA, USA) and Bioanalyzer 2100 system (Agilent Technologies, Santa Clara, CA, USA). Sequencing was subsequently performed on the MGI-SEQ 2000 platform by Frasergen Bioinformatics Co., Ltd. (Wuhan, China) [13].

Differentially expressed genes between sample groups were evaluated using DESeq2 [14]. The false discovery rate (FDR) was used to identify the threshold of the p-value in multiple tests to compute the significance of the differences. Here, only genes with |log_2_ (fold change)| ≥1 and FDR significance score (pad j) < 0.01 were used for subsequent analysis. GO annotation was performed based on the correspondence between the genes in the NCBI GO annotations. The database of this correspondence was obtained from https://ftp.ncbi.nlm.nih.gov/gene/DATA/gene2go.gz. KEGG pathway annotation was performed using BLASTx against plant-specific sequences from the KEGG database. GO and KEGG enrichment analyses were performed using the hypergeometric test, as implemented in the R phyper function [15, 16]. We have got permission to use the KEGG software from the Kanehisa Laboratory (Ref: 220,736).

### Reverse transcription and real-time quantitative polymerase chain reaction (RT-qPCR)

Aliquots (1 µg) of total RNA from each sample were subjected to cDNA synthesis using the HiScript II Q RT SuperMix for qPCR gDNA wiper (Vazyme, Nanjing, China). Primers used for real-time PCR are listed in Additional file 1: Table S1. RT-qPCR was performed with a QuantStudio 3 RT-qPCR System (Applied BioSystems, Foster City, CA, USA) using 2X M5 HiPer SYBR Premix EsTaq (Mei5 Biotechnology, Beijing, China) according to the manufacturer’s recommendations. The expression levels of the target genes were normalized to those of GAPDH.

For SARS-CoV-2 RT-qPCR, 100 ng RNA was used as a template for the amplification of selected genes by RT-qPCR using TransScript^®^ II Probe One-Step RT-qPCR SuperMix (TransGen Biotech, Beijing, China). The SARS-CoV-2 RdRP-positive control plasmid was purchased from Genscript (Nanjing, China). The RT-qPCR system and amplification were performed following the manufacturer’s instructions.

### Western blot analysis

The western blot analysis was performed as described previously [17]. SK-N-SH cells were centrifuged at 12,000 rpm for 10 min at 4 °C to remove insoluble cell debris. The soluble protein concentration in the supernatants was measured using a BCA protein assay kit (NCM Biotech, Suzhou, China). Aliquots from each sample were separated by 12% SDS-PAGE and then transferred to polyvinylidene difluoride membranes. The blots were blocked with 5% bovine serum albumin in Tris-buffered saline containing Tween-20 at room temperature for 2 h. And the blots were cut prior to hybridization with antibodies. Then the blots were incubated overnight at 4 °C with primary antibodies against IL1B, IL6, TNF, Akt, p-Akt, ACTB (Proteintech, Chicago, IL, USA), p38, p-p38, ERK1/2, p-ERK1/2, JNK, p-JNK, mTOR, p-mTOR, p65, and p-p65 (Cell Signaling Technology, Danvers, MA, USA). The blots were subsequently incubated with horseradish peroxidase-conjugated anti-rabbit or anti-mouse IgG (Biodragon, Beijing, China) at 37 °C for 1 h. The densitometry analysis was performed using ImageLab software version 5.2.1 (Bio-Rad, Hercules, CA, USA).

### Immunofluorescence assay

SK-N-SH cells grown in 6-well dishes were fixed with 4% paraformaldehyde for 30 min and then washed three times in PBS. Cells were incubated with the primary rabbit SARS-COV-2 Spike antibody (Abclonal, Wuhan, China) overnight at 4 °C and then incubated with Alexa Fluor 594 goat anti-rabbit antibody (Bioss, Woburn, MA, USA) for another 1 h. Cells were then counterstained with DAPI (US Everbright Inc., Suzhou, China) to visualize the nucleus morphology. Cells were mounted and photographed using BX41 fluorescence microscopy (Olympus, Tokyo, Japan) [18].

### Statistical analysis

Data are expressed as the mean ± standard deviation (SD), and the significance of differences between groups was evaluated using multiple t-tests. A p-value of < 0.05 (*) was considered significant, and p < 0.01 (**) or p < 0.001 (***) were considered extremely significant. Graphs were plotted and analyzed using GraphPad Prism ver. 6.0 (GraphPad Software, La Jolla, CA, USA).

## Results

### SARS-CoV-2 infects SK-N-SH cells

**Fig. 1:**
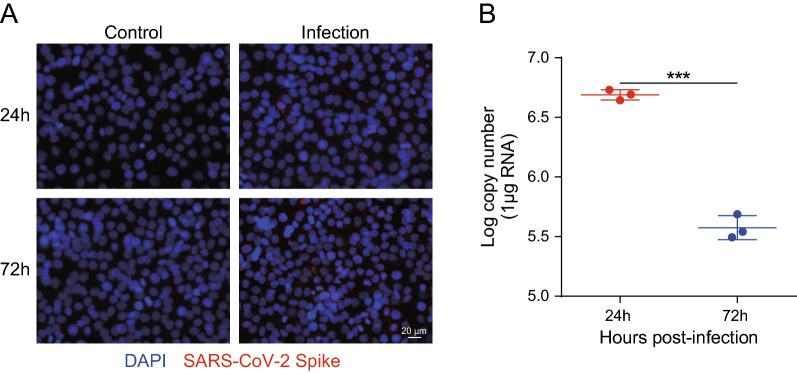
Infection efficiency of SARS-CoV-2 analysis by quantitative real-time polymerase chain reaction (RT-qPCR) and immunofluorescence. **A** Infection efficiency of SARS-CoV-2 was detected using SARS-CoV-2 Spike rabbit antibody by immunofluorescence. Nuclei were stained with DAPI, while SARS-CoV-2 was stained in red. Scale bar, 20 μm. **B** RT-qPCR for the RdRP gene of SARS-CoV-2. Data are expressed as the mean ± standard deviation (SD) from three independent experiments. ***p < 0.001

We sought to determine the level of SARS-CoV-2 replication in infected SK-N-SH cells at 24 and 72 h by RT-qPCR and immunofluorescence. SK-N-SH cells were infected with SARS-CoV-2 at an MOI of 1 and processed for examination. As shown in Fig. 1A, SARS-CoV-2 replicated productively in SK-N-SH cells, as demonstrated by viral antigen staining 24 and 72 h after infection. To confirm this finding, viral RNA was detected using RT-qPCR (Fig. 1B). Viral RNA in the culture maintained a high level at 24 and 72 h post-infection, indicating productive SARS-CoV-2 infection in SK-N-SH cells. Together, these results indicate that SARS-CoV-2 has an infective effect on SK-N-SH cells.

### Identification of differentially expressed mRNAs in SK-N-SH cells in response to SARS-CoV-2

**Fig. 2:**
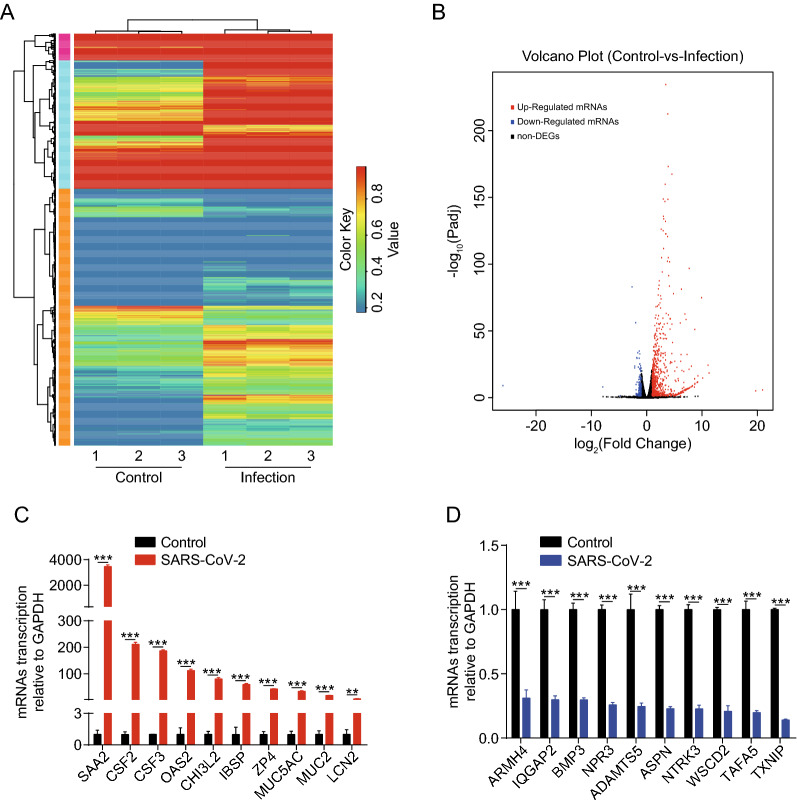
Expression profiling of the changes in mRNAs in SARS-CoV-2-infected SK-N-SH cells. **A** Heat map showing unsupervised clustering of significantly expressed mRNAs from SK-N-SH cells in the SARS-CoV-2 infection groups compared with in the control groups. The expression profiles are displayed with three samples in each group. Red represents high relative expression and blue represents low relative expression. **B** Volcano plot of the upregulated and downregulated mRNAs from SK-N-SH cells in the SARS-CoV-2 infection group compared with in the control group. Increases of ≥ 2-fold or decreases to ≤ 0.5-fold (p < 0.05) were considered statistically significant. **C**, **D** The relative transcription level of 20 mRNAs, including 10 downregulated and 10 upregulated mRNAs, were examined by RT-qPCR in SK-N-SH cells treated with or without SARS-CoV-2 for 72 h. GAPDH was used as the internal reference. Data were presented as the mean ± standard deviation (SD) from three independent experiments. **p < 0.01, ***p < 0.001

To better understand the pathogenic mechanism by which SARS-CoV-2 infects SK-N-SH cells, we conducted a comparative transcriptomic analysis of three uninfected and three infected groups. The heat map and volcano plot revealed the alteration trends of these mRNAs in the cells upon SARS-CoV-2 infection (Fig. 2A and B). We identified 671 upregulated and 149 downregulated mRNAs (showing an increase of ≥ 2-fold or decrease to ≤ 0.5-fold, respectively, p ≤ 0.05) in SARS-CoV-2-infected SK-N-SH cells compared with in uninfected cells (Additional file 2: Table S2). The most significantly upregulated and downregulated mRNAs are listed in Tables 1 and 2, respectively. Next, we used RT-qPCR to verify the results of the differentially altered mRNAs from the sequencing data. To this end, we randomly selected 10 significantly upregulated and 10 significantly downregulated mRNAs from the sequencing data. The results demonstrated that the upregulation (Fig. 2C) or downregulation (Fig. 2D) of the mRNAs were in agreement with the findings of RNA-Seq. Here, our work showed that SARS-CoV-2 infection of SK-N-SH cells caused transcriptional alterations in the mRNA profiles of these cells.

**Table 1:** The most significantly up-regulated mRNAs in SK-N-SH upon infection

Gene symbol	Ensembl gene ID	log2(fold change)	pvalue	qvalue
DAXX	ENSG00000204209	20.94920312	8.40E−08	1.64E−06
PRKAR2B	ENSG00000005249	19.70849114	4.64E−07	7.93E−06
IL1B	ENSG00000125538	11.27229587	4.04E−21	3.19E−19
C3	ENSG00000125730	11.09132179	3.92E−27	4.45E−25
NPHS1	ENSG00000161270	10.13167858	3.27E−17	1.97E−15
CXCL8	ENSG00000169429	9.912163891	2.54E−78	1.33E−75
CSF2	ENSG00000164400	9.254520273	6.43E−19	4.50E−17
CSF3	ENSG00000108342	9.209276009	4.21E−14	1.88E−12
SAA1	ENSG00000173432	9.115529729	1.00E−13	4.32E−12
SAA2	ENSG00000134339	8.723468366	5.53E−13	2.22E−11
CXCL1	ENSG00000163739	8.696454974	3.03E−54	8.37E−52
IBSP	ENSG00000029559	8.689388507	2.50E−12	9.31E−11
MUC5AC	ENSG00000215182	8.646083669	1.23E−16	7.06E−15
CCL5	ENSG00000271503	8.620893187	3.94E−10	1.08E−08
DAZL	ENSG00000092345	8.289859122	3.31E−11	1.07E−09
CHI3L2	ENSG00000064886	8.22848944	8.28E−11	2.53E−09
CXCL6	ENSG00000124875	8.07779969	2.22E−26	2.43E−24
OAS2	ENSG00000111335	8.02626939	3.40E−10	9.40E−09
CXCL10	ENSG00000169245	7.762963407	1.29E−09	3.25E−08
CCL11	ENSG00000172156	7.693464893	2.21E−09	5.44E−08
CXCL2	ENSG00000081041	7.687061845	2.01E−100	1.51E−97
CXCL3	ENSG00000163734	7.657960595	5.81E−24	5.49E−22
LCN2	ENSG00000148346	7.571353618	4.88E−09	1.15E−07
ZP4	ENSG00000116996	7.46003184	1.01E−08	2.28E−07
MUC2	ENSG00000198788	7.397325048	1.69E−08	3.71E−07
TRIM63	ENSG00000158022	7.318006714	5.26E−09	1.24E−07
SELE	ENSG00000007908	7.181187414	5.77E−08	1.17E−06
HSD11B1	ENSG00000117594	7.120788422	8.04E−62	2.86E−59
CCL20	ENSG00000115009	7.094282144	1.98E−08	4.29E−07
ICAM4	ENSG00000105371	6.980521281	3.59E−08	7.50E−07

**Table 2:** The most significantly down-regulated mRNAs in SK-N-SH upon infection

Gene symbol	Ensembl Gene ID	log2 (fold change)	pvalue	qvalue
ART1	ENSG00000129744	− 25.97257384	2.97E−11	9.65E−10
H3C14	ENSG00000203811	− 7.937669315	3.29E−10	9.13E−09
EPHB6	ENSG00000106123	− 4.767816987	0.007234618	0.04103239
TXNIP	ENSG00000265972	− 2.641537556	1.59E−86	9.85E−84
ASPN	ENSG00000106819	− 2.166774086	4.96E−25	4.96E−23
NPR3	ENSG00000113389	− 1.980348765	2.11E−59	7.38E−57
NTRK3	ENSG00000140538	− 1.917568906	3.93E−12	1.41E−10
BMP3	ENSG00000152785	− 1.917323176	6.36E−08	1.27E−06
TAFA5	ENSG00000219438	− 1.905480933	6.07E−05	0.000656346
novel.9455	–	− 1.887402389	0.000170931	0.00164349
ARHGAP11A	ENSG00000198826	− 1.882496615	0.000357935	0.003121564
ARMH4	ENSG00000139971	− 1.874225841	0.001202566	0.008838879
IQGAP2	ENSG00000145703	− 1.810460641	7.72E−33	1.11E−30
ALKAL2	ENSG00000189292	− 1.747772254	0.00483489	0.029334243
WSCD2	ENSG00000075035	− 1.742410707	5.02E−14	2.23E−12
SLITRK1	ENSG00000178235	− 1.733790354	0.000185733	0.001773628
ADAMTS5	ENSG00000154736	− 1.732977261	1.93E−32	2.76E−30
DHCR24	ENSG00000116133	− 1.599309414	1.25E−36	2.11E−34
ZNF488	ENSG00000265763	− 1.581696732	0.000498581	0.004161249
NEUROG2	ENSG00000178403	− 1.568382027	0.001483324	0.01058027
SARDH	ENSG00000123453	− 1.521102529	1.16E−25	1.21E−23
novel.19946	–	− 1.480268427	4.46E−07	7.64E−06
LOC105379752	–	− 1.476954229	0.004282749	0.026536869
C11orf87	ENSG00000185742	− 1.454286156	3.43E−07	5.97E−06
CALB1	ENSG00000104327	− 1.453649367	6.96E−05	0.000738503
FRMPD1	ENSG00000070601	− 1.428168219	1.95E−05	0.000239099
RAG2	ENSG00000175097	− 1.426357278	4.38E−16	2.39E−14
EMILIN3	ENSG00000183798	− 1.422461403	0.006872679	0.039366988
novel.8416	–	− 1.421986068	0.000205758	0.001943637
ADARB2	ENSG00000185736	− 1.420609303	1.53E−13	6.56E−12

### Bioinformatics analysis of the altered mRNAs

**Fig. 3:**
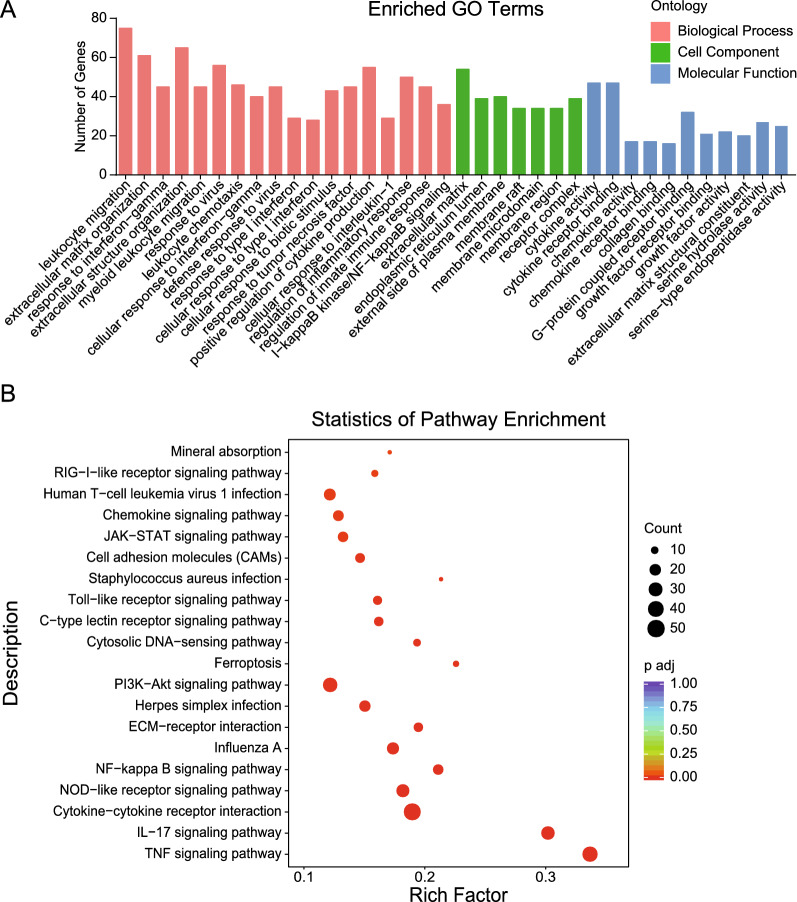
Summary of the Gene Ontology (GO) and Kyoto Encyclopedia of Genes and Genomes (KEGG) pathway analysis for the differentially expressed mRNAs. **A** GO analysis of the differentially expressed mRNAs. The x-axis represents the name of the enrichment pathway. The y-axis represents the targeted gene numbers corresponding to the GO terms. **B** KEGG analysis of the 20 most enriched pathways. The coloring of the p-values indicates the significance of the rich factor. Circles indicate the target genes that are involved, and their sizes are proportional to the number of genes. The x-axis represents the rich factor. The y-axis represents the name of the enrichment pathway

To better understand the pathogenic mechanism by which SARS-CoV-2 infects SK-N-SH cells, bioinformatics approaches were used to analyze the potential function of 820 differentially expressed mRNAs in SARS-CoV-2-infected SK-N-SH cells. These mRNAs were assigned to different categories of “biological processes,” “cellular components,” and “molecular functions.” Within the biological processes category, these mRNAs were mainly involved in leukocyte migration, extracellular matrix organization, response to interferon-gamma, response to virus, positive regulation of cytokine production, and regulation of inflammatory response. In the cellular components category, these mRNAs were mainly divided into extracellular matrix, endoplasmic reticulum lumen, external side of plasma membrane, membrane raft, and receptor complex. Within the molecular function category, these altered mRNAs were mainly associated with cytokine activity, cytokine receptor binding, chemokine activity, chemokine receptor binding, G-protein coupled receptor binding, growth factor activity, and serine hydrolase activity (Fig. 3A). Moreover, the signaling pathways enriched by these 820 significantly changed mRNAs were determined using KEGG analysis. The results revealed that several canonical signaling pathways were significantly enriched, some of which have already been shown to be involved in the inflammatory response (e.g., cytokine-cytokine receptor interaction, NOD-like receptor signaling pathway, Toll-like receptor signaling pathway, NF-κB signaling pathway, chemokine signaling pathway, IL-17 signaling pathway, *Staphylococcus aureus* infection, and TNF signaling pathway), and pathogenic microbial infection (e.g., influenza A, herpes simplex infection, TNF signaling pathway, and human T cell leukemia virus 1 infection) (Fig. 3B). These results effectively revealed that the signaling pathways participated in the mechanism by which SARS-CoV-2 induces a severe CNS inflammatory response.

### SARS-CoV-2 infection induces high levels of inflammatory response in SK-N-SH cells

**Fig. 4:**
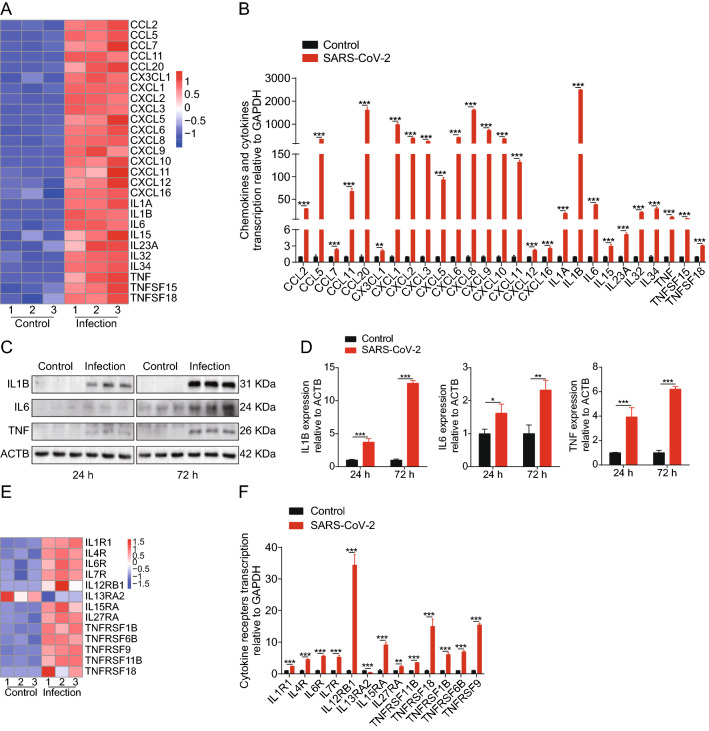
SARS-CoV-2 infection of SK-N-SH cells induced a severe inflammatory response. **A** Heat maps depicting virally regulated cytokines and chemokines upon SARS-CoV-2 infection in SK-N-SH cells. Colored bar represents expressive abundance of log2 transformed values. **B** RT-qPCR analysis of cytokines and chemokines transcription in SK-N-SH cells upon SARS-CoV-2 infection. GAPDH was used as the internal reference. Data were presented as the mean ± standard deviation (SD) from three independent experiments. **p < 0.01, ***p < 0.001. **C** Western blot analysis of IL1B, IL6, and TNF in cell lysates of SK-N-SH cells after infection 24 and 72 h. ACTB was used as the loading control. **D** Densitometry was performed to analyze differences among the samples. *p < 0.05, **p < 0.01, ***p < 0.001. **E** Heat maps depicting virally regulated cytokine receptors upon SARS-CoV-2 infection in SK-N-SH cells. Colored bar represents expressive abundance of log2 transformed values. **F** RT-qPCR analysis of cytokine receptors transcription in SK-N-SH cells upon SARS-CoV-2 infection. GAPDH was used as the internal reference. Data were presented as the mean ± SD from three independent experiments. **p < 0.01, ***p < 0.001

To date, studies have shown elevated levels of neuroinflammatory markers in the plasma and cerebrospinal fluid of COVID-19 patients [19]. Here, we verified the induction of cytokines, chemokines, and cytokine receptors in SARS-CoV-2-infected SK-N-SH cells. As shown in the heat map in Fig. 4A, we analyzed the induction of cytokines and chemokines in SK-N-SH cells upon SARS-CoV-2 infection and found that SARS-CoV-2 infection could significantly induce the upregulation of multiple cytokines (e.g., IL1B, IL6, IL32, IL34, TNF, etc.) and chemokines (e.g., CCL5, CCL20, CXCL1, CXCL2, CXCL3, etc.). Next, we employed RT-qPCR to verify the results of the significantly upregulated cytokines and chemokines from the RNA-Seq data. The qPCR results of 27 differentially expressed cytokines and chemokines were consistent with the RNA-Seq data (Fig. 4B). Moreover, we found that the protein levels of IL1B, IL6, and TNF showed a significant and time-dependent increase accompanying infection (Fig. 4C and D). Since the TNF signaling pathway, IL17 signaling pathway, and cytokine-cytokine receptor interaction were enriched in the KEGG analysis, we then summarized the expression of cytokine receptors. As shown in the heat map in Fig. 4E a total of 13 cytokine receptors were significantly altered in SK-N-SH cells during SARS-CoV-2 challenge. RT-qPCR assays were performed to confirm the overexpression of these cytokine receptors in SARS-CoV-2-challenged SK-N-SH cells. As observed in Fig. 4F, the transcription of all 13 cytokine receptors significantly changed upon SARS-CoV-2 infection. Taken together, these results indicate that SARS-CoV-2 infection of SK-N-SH cells can induce significant production of proinflammatory cytokines and cytokine receptors.

### NF-κB, p38, and akt signaling pathways are involved in SARS-CoV-2 infection of SK-N-SH cells

**Fig. 5:**
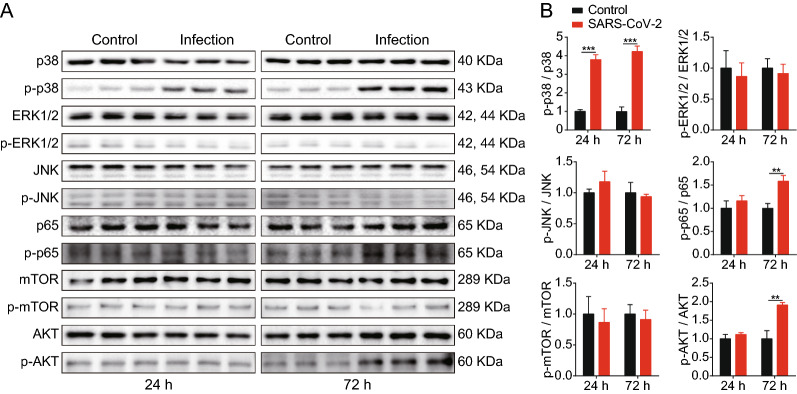
Akt, p65, and p38 signaling were activated in SK-N-SH cells upon SARS-CoV-2 infection. **A** Phosphorylation of p38, ERK1/2, JNK, p65, mTOR, and Akt in SK-N-SH cells upon challenge of SARS-CoV-2 at 24 and 72 h were determined by western blot. **B** Densitometry was performed to analyze differences among the samples. **p < 0.01, ***p < 0.001

Based on our KEGG and GO signaling pathway predictions, we next investigated the activation of NF-κB, MAPK (p38, JNK, and ERK1/2), and Akt pathways in SK-N-SH cells in response to SARS-CoV-2. The results showed that the phosphorylation of p65, p38, and Akt significantly increased in response to SARS-CoV-2 (Fig. 5A and B) in a time-dependent manner, indicating the activation of the NF-κB, p38, and Akt pathways in SK-N-SH cells upon SARS-CoV-2 challenge. Conversely, it was found that the phosphorylation of mTOR, JNK, and ERK1/2 was not significantly altered in SK-N-SH cells 24 and 72 h post-challenge compared with in uninfected cells. These results indicate that the NF-κB, p38, and Akt signaling pathways are activated and participate in SARS-CoV-2 infection.

## Discussion

**Fig. 6:**
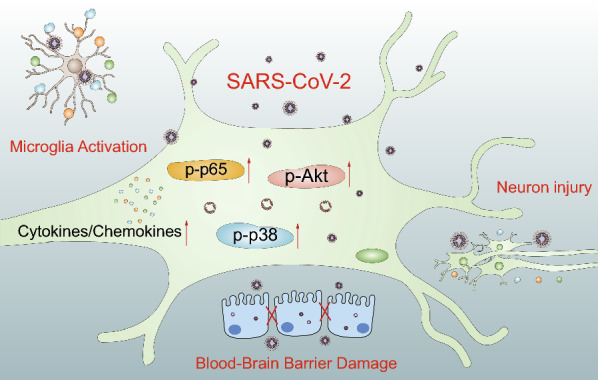
Schematic representation of SARS-CoV-2-induced neuron injury and neuroinflammation

Increasing evidence indicates that SARS-CoV-2 may invade the CNS and cause neurological disorders [20]. The most common neurological manifestation reported by COVID-19 patients is the loss of taste and smell, suggesting that SARS-CoV-2 infection might also affect cells within the CNS [21]. To extend the existing knowledge and to provide further evidence for the invasion of SARS-CoV-2 within CNS cells, we applied RNA-Seq to identify the differentially expressed mRNAs in SK-N-SH cells in response to SARS-CoV-2 infection. The transcription level of 820 mRNAs significantly changed in response to infection, among which 671 mRNAs were significantly increased and 149 mRNAs were significantly decreased. In addition, GO and KEGG pathway analysis indicated that the JAK-STAT, PI3K-Akt, NF-κB, IL17, and TNF signaling pathways might play important roles in the development of SARS-CoV-2 infection in SK-N-SH cells. Our western blot data also demonstrated that the phosphorylation of p65, p38, and Akt significantly increased in SARS-CoV-2-infected SK-N-SH cells in a time-dependent manner, indicating that the NF-κB, p38, and Akt signaling pathways play an important role in SARS-CoV-2 infection. We further showed that SARS-CoV-2 caused a sharp increase in the production of cytokines (e.g., IL1B, IL6, IL34, etc.) and chemokines (e.g., CCL5, CCL20, CXCL1, CXCL8, etc.) in SK-N-SH cells, which may largely promote the development of CNS inflammatory response (Fig. 6). To the best of our knowledge, this is the first study on the differential induction of host mRNAs in SK-N-SH cells by SARS-CoV-2, providing a theoretical basis for future studies on SARS-CoV-2 infection.

A retrospective case series demonstrated that the neurological symptoms induced by SARS-CoV-2-infection include CNS symptoms (e.g., headache, dizziness, impaired consciousness, ataxia, acute cerebrovascular disease, and epilepsy), peripheral nervous system symptoms (such as hyposmia, hypogeusia, hypopsia, and neuralgia), and skeletal muscle symptoms [22–24]. Meanwhile, accumulating tissue-based studies analyzing CNS alterations in fatal COVID-19 have provided evidence of histopathological changes occurring in COVID-19. These changes involve hypoxia-related pathology, including CNS infarction due to cerebral thromboembolism and signs of a CNS-intrinsic myeloid cell response [25, 26]; this finding indicates the presence of SARS-CoV-2 in the CNS [27, 28]. In general, hematogenous and neuronal retrograde dissemination are two principal routes for viral entry into the CNS [29]. It has also been found that SARS-CoV-2 can invade leukocytes for dissemination into the CNS [30]. In our other work, we demonstrated that SARS-CoV-2 can infect endothelial cells of the blood–brain barrier (BBB) and damage its integrity, thereby allowing direct passage across the BBB into the CNS [31]. As another strategy to enter the CNS, some neurotropic viruses such as rabies viruses infect neurons in the periphery and use the axonal transport machinery to cross the host BBB [32]. The latest publication has established the neuroinvasion ability of SARS-CoV-2 and provides the first data showing that SARS-CoV-2 could directly infect induced pluripotent stem cell-derived human neural progenitor cells. Furthermore, extensive viral replication and viral particles were detected in the neurospheres and brain organoids with SARS-CoV-2 infection [33, 34]. However, in our work, data obtained via RT-qPCR and immunofluorescence only supported the notion that SK-N-SH cells allow SARS-CoV-2 infection but do not support viral replication. The above differences may be due to the inconsistency of cell models.

SARS-CoV-2 infection has been shown to be associated with high levels of chemokines and cytokines, including IL1B, IL2, IL6, IL7, IL8, IL10, IL17, IFNG, CXCL10, CCL2, CCL3, CCL7, and TNF, in a phenomenon known as cytokine storm; high levels of these proinflammatory cytokines have been linked to poor outcomes [35–38]. Complicated cases of COVID-19 exhibit higher levels of proinflammatory cytokines, such as IL6 and IFNG, making them more susceptible to neurological complications [39]. Studies on postmortem cases indicate that lymphocytes and monocytes infiltrate the vessel walls in the brain, exacerbating neuronal degeneration and demyelination [40]. In addition, enhancement of chemokines and cytokines might lead to BBB dysfunction, which is a common feature of many infectious CNS diseases [41]. Brain microvascular endothelial cells (BMECs) are the most prominent cell type of the BBB [42] and are characterized by the presence of several tight junction (TJ) proteins [43]. Decrease or redistribution of tight junction proteins could lead to increased BBB permeability, which is an important indicator of BBB damage [44]. For example, CCL2 has been shown to reduce the expression of TJP1, TJP2, OCLN, and CLDN5 in HIV-1-infected BMECs through Rho kinase signaling [45]. It has been reported that a recombinant rabies virus expressing a chemokine (CXCL10 and CCL5) enhanced BBB permeability in mice and reduced the expression of TJ proteins TJP1, OCLN, and CLDN5 in BMECs [46]. In a study of herpes simplex virus-1 encephalitis, CXCL1 is produced by astrocytes in response to viruses and by astrocytes and neurons in response to IL1A; CXCL1 is the critical ligand required for neutrophil transendothelial migration, which correlates with BBB breakdown [47]. In addition, recent studies suggest that BBB integrity is disrupted, and lymphocytic pleocytosis and white blood cell infiltration into the CNS occur in these patients [48]. Indeed, our data showed that SARS-CoV-2 infection of SK-N-SH cells can significantly induce high levels of chemokines and cytokines. These include CCL2, CCL5, CCL7, CCL11, CCL20, CXCL1, CXCL2, CXCL3, CXCL5, CXCL6, CXCL8, CXCL9, CXCL10, CXCL11, CXCL12, CXCL16, IL1A, IL1B, IL6, IL15, IL23A, IL32, IL34, and TNF.

## Conclusions

In our study, using a high-throughput RNA-Seq approach, we compared and analyzed the significantly altered mRNAs in SK-N-SH cells infected with or without SARS-CoV-2. A total of 820 differentially expressed mRNAs were identified as the target genes in SK-N-SH cells upon infection with SARS-CoV-2. Our data revealed for the first time the transcription profiles of differentially expressed cytokines and chemokines in neuronal cells infected by SARS-CoV-2, while also showing that the NF-κB, Akt, and p38 signaling pathways are involved in the infection process. Comparing and profiling these differentially expressed mRNAs in SK-N-SH cells in response to SARS-CoV-2 should lead to further research on host responses against SARS-CoV-2 and aid in developing more targets for better prevention and therapeutic control of SARS-CoV-2 infection.

## Supplementary Information

**Additional file 1**: **Table S1. **Primers used for RT-qPCR in this study.

**Additional file 2**: **Table S2. **Differentially expressed mRNAs (Control vs Infection).

## Data Availability

The data that support the findings of this study are available from the corresponding author upon reasonable request. The datasets generated and/or analysed during the current study are available in the GEO repository (Accession Number PRJNA663977, https://www.ncbi.nlm.nih.gov/bioproject/PRJNA663977).

## Abbreviations

SARS-CoV-2: Severe acute respiratory syndrome coronavirus 2
BBB: Blood–brain barrier
CNS: Central nervous system
SARS: Severe acute respiratory syndrome
MERS: Middle East respiratory syndrome
COVID-19: Coronavirus disease 19
ARDS: Acute respiratory distress syndrome
ACE2: Angiotensin-converting enzyme 2
RNA-seq: RNA sequencing
MEM: Minimum essential medium
FBS: Fetal bovine serum
MOI: Multiplicity of infection
PBS: Phosphate-buffered saline
NCBI: National center for biotechnology information
FDR: False discovery rate
KEGG: Kyoto Encyclopedia of Genes and Genomes
GO: Gene ontology
RT-qPCR: Reverse transcription and real-time quantitative polymerase chain reaction
cDNA: Complementary DNA
SD: Standard deviation
